# Electroacupuncture Attenuates Intestinal Barrier Disruption via the α7nAChR-Mediated HO-1/p38 MAPK/NF-κB Pathway in a Mouse Model of Metabolic Dysfunction-Associated Fatty Liver Disease: A Randomized, Single-Blind, Controlled Trial

**DOI:** 10.3390/biomedicines13040802

**Published:** 2025-03-27

**Authors:** Xiao Wang, Jiasen Sun, Peng Wang, Yimin Zhang, Jiuyang Chang, Zhijun Duan

**Affiliations:** 1 Department of Gastroenterology, The First Affiliated Hospital of Dalian Medical University, Dalian 116011, China; shallwang_med@163.com (X.W.); xhbksjs@163.com (J.S.); wangpengm0801@126.com (P.W.); yiminzhang2024@163.com (Y.Z.); 2 Laboratory of Integrated Medicine, The First Affiliated Hospital of Dalian Medical University, Dalian 116011, China; 3 Dalian Central Laboratory of Integrative Neuro-Gastrointestinal Dynamics and Metabolism Related Diseases Prevention and Treatment, Dalian 116011, China; 4 Department of Cardiology, The First Affiliated Hospital of Dalian Medical University, Dalian 116011, China; jychang0424@gmail.com

**Keywords:** electroacupuncture, MAFLD, intestinal barrier, α7nAChR, HO-1

## Abstract

**Background:** Gut barrier integrity plays a crucial role in the pathogenesis of metabolic dysfunction-associated fatty liver disease (MAFLD). Electroacupuncture (EA) at ST-36 can ameliorate inflammatory responses via stimulating the α7 nicotinic acetylcholine receptor (α7nAChR), but whether EA is effective in preserving the intestinal barrier of MAFLD has not been exactly illustrated. This investigation explored potential protection mechanisms of EA at ST-36 targeting the dismantled gut barrier in MAFLD. **Methods:** C57BL/6 mice were randomly allocated into several subgroups: control (CON), high-fat diet (HFD), HFD with EA, HFD with EA and α7nAChR inhibitor α-BGT, and HFD with EA and intestinal HO-1 knockout (KO). Body weight, liver weight, visceral fat index, and histopathological examination of the liver and the intestine were determined. Serum biological indexes were evaluated through corresponding kits. Furthermore, the expressions of HO-1, α7nAChR, gut barrier-associated proteins, and the molecular mechanisms in intestinal tissues were assessed via Western blot, RT-qPCR, immunohistology, or immunofluorescence examination. **Results:** EA treatment decreased body weight, liver weight, and visceral fat index gain and mitigated liver function injury and abnormal lipid indexes, exhibiting less severity of hepatic steatosis, fibrosis, and inflammation responses of MAFLD. Lower gut permeability, less intestinal epithelial disruption, and upregulation of tight junction proteins after EA suggested the protective effects in attenuating intestinal epithelial barrier dysfunction. These protective effects were abolished by α-BGT or intestinal HO-1 deletion. Mechanistically, EA markedly enriched α7nAChR and HO-1 expression and mitigated phosphorylated p38 MAPK/NF-κB activation, which was lost in α-BGT or HO-1 KO treatment. **Conclusions:** The protective effects of EA at ST-36 in the pathogenesis of MAFLD may be attributed to the preserved intestinal barrier, thereby alleviating systemic inflammatory responses and preventing subsequent liver hits, where the α7nAChR-mediated HO-1/p38 MAPK/NF-κB pathway was crucial to maintain homeostasis.

## 1. Introduction

Metabolic dysfunction-associated fatty liver disease (MAFLD) is a heterogeneous disorder featuring various spectrums of hepatic steatosis and metabolic abnormalities, impacting around one-quarter of the global population and raising the risk of several dysfunctions, such as insulin resistance, chronic renal failure, and coronary atherosclerotic disorders [1,2]. Emerging evidence has highlighted intestinal barrier impairment as a key pathogenic driver in MAFLD [3]. Specifically, disrupted intestinal barrier integrity facilitates aberrant translocation of gut-derived products (e.g., endotoxin) and pro-inflammatory cytokines via the portal circulation, triggering endotoxemia, systemic inflammation, and liver attacks, further exacerbating MAFLD progression [3,4,5]. Thus, preserving a multifunctional gut barrier may be a promising therapeutic strategy for maintaining metabolic homeostasis in MAFLD.

Electroacupuncture (EA), a traditional treasure with painlessness, safety, and effectiveness features, has demonstrated efficacious modulation in several biological processes, such as oxidative stress, immunity homeostasis, inflammatory responses, and gut barrier disruption [6,7,8,9]. Regulatory mechanisms of EA involve the activation of the cholinergic anti-inflammatory pathway (CAP) through vagus nerve stimulation [10,11]. The α7 nicotinic acetylcholine receptor (α7nAChR), an essential mediator in CAP, can mitigate systemic inflammation by suppressing pro-inflammatory cascades, such as nuclear factor-kappa B (NF-κB) [12,13]. However, whether pleiotropic EA could ameliorate intestinal barrier impairment in MAFLD via α7nAChR-dependent mechanisms requires further exploration.

Heme oxygenase-1 (HO-1) is a multifunctional isozyme that exerts anti-oxidant and anti-inflammatory properties under pro-oxidant stimuli [14,15]. Several studies have indicated that HO-1 is responsible for upregulating the expression of intestinal tight junction proteins (e.g., Occludin), alleviating inflammation reactions, and preserving intestinal barrier permeability, suggesting key contributions of HO-1 in maintaining intestinal barrier integrity [16,17,18,19]. Furthermore, the protective effects of HO-1 involve the regulation of several downstream effectors. Specifically, HO-1 can suppress p38 mitogen-activated protein kinase (MAPK) activation, which further inhibits the phosphorylation of downstream molecules such as NF-κB [14]. Notably, evidence demonstrates that both EA intervention and pharmacological α7nAChR activation can preserve the disrupted intestinal barrier in the cholestatic liver injury model via enhancing HO-1 signaling [12,16].

In this current research, we aim to explore the therapeutic effects and underlying mechanisms of EA on gut barrier dysfunction in the pathogenesis of MAFLD, which may provide a novel basis for the clinical application of EA in MAFLD treatment.

## 2. Materials and Methods

### 2.1. Animals

Male C57BL/6 mice (aged about 7 weeks, weighing 19–21g; Viewsolid Biotechnology, Beijing, China) were utilized. The VillinCreHmox1floxp/floxp transgenic mice line was generated through breeding Vil-linCre transgenic mice with Hmox1floxp/floxp mice carrying LoxP sites flanking Hmox-1 exon 2, which was conducted by Viewsolid Biotechnology (Beijing, China). Mice were placed in ventilated cages of the SPF Animal Experiment Center under controlled environmental temperature and specific humidity with a regular 12/12 h light-dark cycle. Mice had unlimited availability of pellet food and clean water, with 1 week of acclimatization before the experiment. Animal experiments were approved by the Institutional Animal Ethics Committee of Dalian Medical University (approval number: AEE21064).

### 2.2. Animal Grouping and Interventions

**Figure 1:**
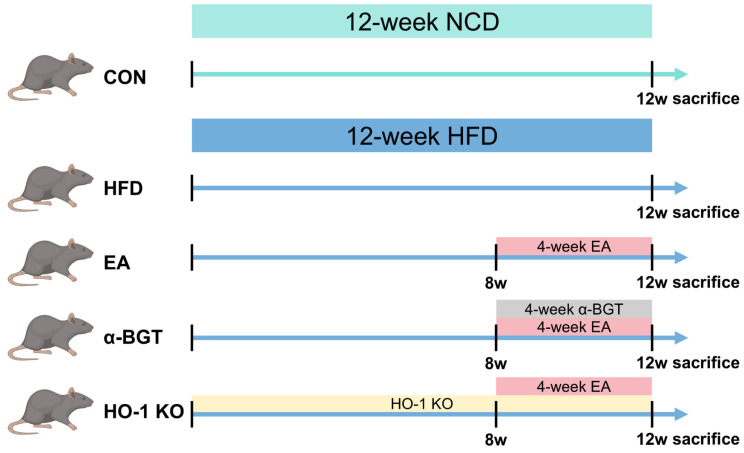
Schematic presentation of the experimental procedures.

Mice were allocated randomly into five groups according to a random number table shown in Figure 1: control (CON), high-fat diet (HFD)-induced MAFLD (HFD), HFD + EA (EA), HFD + EA + a selective α7nAChR inhibitor α-bungarotoxin (α-BGT), and HFD + EA + intestine-specific HO-1 knockout (HO-1 KO) (*n* = 6 per group). The CON group had access to a normal chow diet (NCD) composed of 20% protein, 10% fat, and 70% carbohydrates (kcal%). The last four groups were supplied with a consecutive 12-week HFD to establish the MAFLD model. The HFD comprised 20% protein, 60% fat, and 20% carbohydrates (kcal%). In the last three groups, EA stimulation was conducted under specific parameters of the 45-min stimulation along with a 2mA electric current amplitude and a 50 Hz frequency on alternate days for 4 weeks by an SNM- FDC01 Stimulator (Ningbo, China). Briefly, electrical needles (0.18 × 7 mm) were inserted perpendicularly at ST36 (4 mm away from the knee and 2mm lateral around the anterior tibial tuberosity). In the α-BGT group, α-BGT pre-treatment (1 μg/kg) was intraperitoneally provided 30 min before each EA stimulation.

Finally, the blood, liver, intestine, and abdominal visceral fat (including perirenal, epididymal, and mesenteric fat) were collected for subsequent research. All laboratory workers were blind to group allocation throughout the experiments and analysis.

### 2.3. Histological Analysis

Pathological liver and intestine specimens with 4% paraformaldehyde (PFA) were embedded in paraffin blocks for further slicing into 3–4 μm segments and stained by hematoxylin and eosin (H&E) for the assessment of impairment severity and inflammatory cellular infiltration. Hepatic steatosis and fibrogenesis were evaluated through Oil Red O and Sirius Red staining under the manufacturer’s instructions. The MAFLD activity score was dependent on three different parameters: lobular inflammatory reaction, hepatic steatosis, and hepatocyte ballooning.

### 2.4. Biochemical Measurement

The serum concentrations of aspartate transaminase (AST), alanine aminotransferase (ALT), total cholesterol (TC), and triglycerides (TG) were qualified using corresponding reagent assays (Nanjing Jiancheng Bioengineering Institute, Nanjing, China). The serum contents of interleukin-6 (IL-6), interleukin-10 (IL-10), interleukin-1β (IL-1β), tumor necrosis factor-α (TNF-α), D-lactate (D-Lac), and endotoxin, along with the intestinal levels of IL-10, were evaluated via related enzyme-linked immunosorbent assay (ELISA) kits (Elabscience, Wuhan, China; Lengton Technology, Shanghai, China).

### 2.5. Analysis of Real-Time Quantitative PCR

RNA was extracted utilizing TRIzol (Accurate Biology, Changsha, China), then transformed inversely into cDNA utilizing the Evo M-MLV Reverse Transcription Mix Kit (Accurate Biology, Changsha, China). Synthesized cDNA was subsequently employed for RT-qPCR utilizing SYBR Green Premix Pro Taq HS qPCR Kit II (Accurate Biology, Changsha, China) on a BIOER System. Specific primers are exhibited in Table 1. Relative quantitative contents in target genes were determined by 2^−ΔΔCt^.

### 2.6. Assessment of Western Blot

The extraction and qualification of total intestinal proteins in different groups were conducted using the RIPA and bicinchoninic acid (BCA) assay kit (Solarbio, Beijing, China). Lysate protein samples were loaded into each lane of 8–12% sodium dodecyl sulfate-polyacrylamide gel electrophoresis (SDS-PAGE) and transferred to polyvinylidene difluoride membranes. After the blockage in 5% skimmed milk (Solarbio, Beijing, China), membranes experienced first incubation with primary antibodies at 4 °C overnight. The utilization of primary antibodies included zonula occludens-1 (ZO-1, AF5145, Affinity, Changzhou, China), Occludin (27260-1-AP, Proteintech, Rosemont, IL, USA), Claudin-1 (ab15098, Abcam, Cambridge, MA, USA), α7nAChR (A1588, ABclonal, Wuhan, China), HO-1 (10701-1-AP, Proteintech, Rosemont, IL, USA), phospho-NF-kB p65 (AF2006, Affinity, Changzhou, China), NF-kB p65 (AF5006, Affinity, Changzhou, China), phospho-p38 MAPK (AP0526, ABclonal, Wuhan, China), p38 MAPK (A14401, ABclonal, Wuhan, China), and β-actin (AC026, ABclonal, Wuhan, China). After washing, the strips were further infiltrated in the secondary antibody (AS014, ABclonal, Wuhan, China) for 1 h at indoor temperature. Strip visualization was performed using a chemiluminescence imaging system (Tanon-5200, Shanghai, China) with a chemiluminescent reagent (Tanon, Shanghai, China). In comparison to the internal reference, the relative amount of expression in target proteins was analyzed and qualified with ImageJ 1.52a software.

### 2.7. Immunohistochemistry and Immunofluorescence Assay

Paraffin-embedded liver and intestine specimens with a thickness of 3–4 μm were utilized. The dewaxing and hydration of the paraffin sections were performed in various concentration gradients of xylene and ethanol. After the function of 3% hydrogen peroxide, the slices were placed for 15 min at 96–98 °C.

During the process of immunohistochemistry staining, after the blockage in 5% bovine serum albumin (BSA, Solarbio, Beijing, China), slices were incubated with primary antibody against α-SMA (14395-1-AP, Proteintech, Rosemont, IL, USA), phospho-NF-kB p65 (AF2006, Affinity, Changzhou, China), and HO-1 (10701-1-AP, Proteintech, Rosemont, IL, USA) at 4 °C. After reheating and washing, slices then underwent respective incubation with secondary antibodies (GB23303, Servicebio, Wuhan, China) and a color rendering agent of 3, 3-diaminobenzidine working solution. After repeated washing, counterstain with hematoxylin and dehydration were applied. The immunohistochemistry results of these sections were observed microscopically.

During the process of immunofluorescence assay, after the blockage in 5% BSA, sections were added into primary antibodies against ZO-1 (AF5145, Affinity, Changzhou, China), Occludin (27260-1-AP, Proteintech, Rosemont, IL, USA), Claudin-1 (ab15098, Abcam, Cambridge, MA, USA), and phospho-p38 MAPK (AP0526, ABclonal, Wuhan, China) for overnight incubation at 4 °C. Then, sections underwent respective incubation with secondary antibodies and a DAPI working solution. The immunofluorescence results of these sections were also observed microscopically.

### 2.8. Statistical Analysis

Experimental data were presented with the mean ± standard error. A comparison of the two groups was shown using a two-tailed student *t*-test. Furthermore, a comparison of several groups was shown following the one-way analysis of variance (ANOVA). *p*-value < 0.05 suggested the existence of statistical differences. In addition, GraphPad Prism 8.0 software was utilized for the analysis and graphic presentation of the experiment results.

## 3. Results

### 3.1. EA Alleviated Hepatic Steatosis in MAFLD

**Figure 2:**
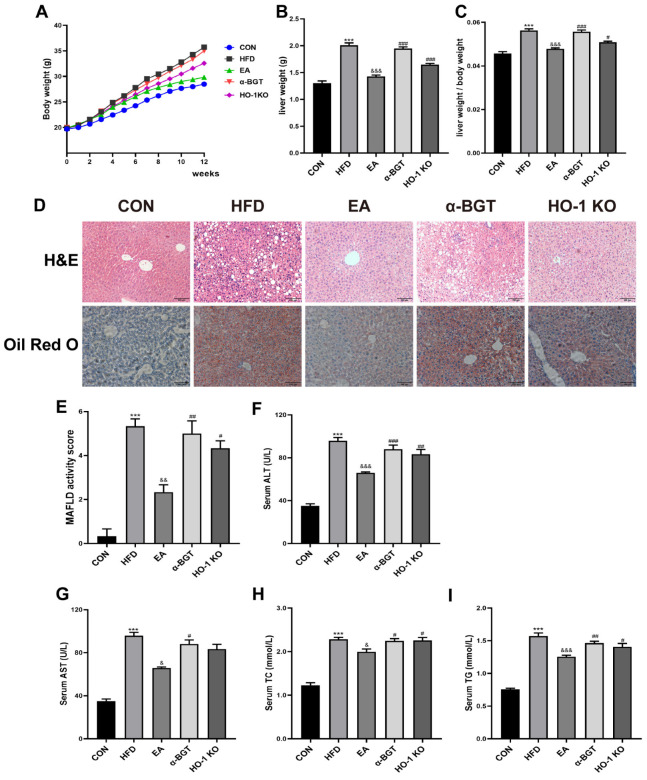
EA alleviated hepatic steatosis in MAFLD. (**A**–**C**) Marked changes in the weight of the body and liver, along with the liver-to-body weight ratio, were measured in MAFLD under exposure to EA. (**D**) H&E and Oil Red O staining were utilized for liver pathological alterations expressed in representative images (scale bar = 100 μm). (**E**) MAFLD activity score was analyzed in different groups. (**F**–**I**) Serum ALT, AST, TC, and TG contents were validated in each group. Data were obtained from at least three independent assays in several groups of mice. *** *p* < 0.001 vs. CON. ^&^ *p* < 0.05, ^&&^ *p* < 0.01, and ^&&&^ *p* < 0.001 vs. HFD. ^#^ *p* < 0.05, ^##^ *p* < 0.01, and ^###^ *p* < 0.001 vs. EA.

We first evaluated whether EA played a beneficial role in relieving hepatic steatosis in MAFLD. In comparison with the CON group, the weight of the body and liver, along with the liver-to-body weight ratio and visceral fat index, was notably increased in the HFD group while reduced under EA stimulation. Meanwhile, improvement in the above indexes was reversed after exposure to α-BGT and HO-1 KO (Table 2; Figure 2A–C). Additionally, as displayed in Figure 2D,E, pathological clues from liver H&E and Oil Red O showed that HFD induced aberrant accumulation of lipid deposits emerging as numerous sizes of vesicular steatosis and infiltrated inflammatory cells, along with a higher histopathological score in the HFD group, indicating the successful induction of MAFLD. Moreover, EA showed protective effectiveness on pathological liver damage, which worsened upon α7nAChR inhibition or HO-1 KO, indicating that α7nAChR and HO-1 exerted a prominent role during EA stimulation in MAFLD. Consistent with the histopathology results, serum concentrations of pathological indicators such as ALT, AST, TC, and TG augmented after HFD, reflecting partial impairment of liver function and lipid metabolism (Figure 2F,G). A reduction in the abovementioned indexes emerged in the EA group, with a worsening tendency in the α-BGT and HO-1 KO group, but the AST levels of EA and HO-1 KO groups did not vary statistically. Collectively, it indicated that EA could relieve HFD-induced liver damage and lipid metabolism disturbance, which worsened after the intervention of α-BGT or HO-1 KO.

### 3.2. EA Ameliorated Liver Fibrosis in MAFLD

**Figure 3:**
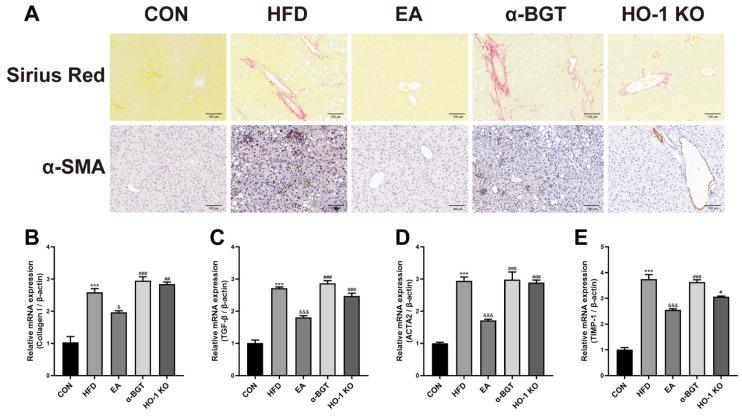
EA ameliorated the severity of liver fibrosis in MAFLD. (**A**) Liver histological examination with Sirius Red staining and α-SMA immunohistochemistry assay was utilized for pathological alterations in the liver expressed in representative images (scale bar = 100 μm). (**B**–**E**) The mRNA contents of several fibrogenic factors in the liver, such as *collagen I*, *TGF-β*, *ACTA2*, and *TIMP-1*, were measured via RT-qPCR assay. Data were obtained from at least three independent assays in several groups of mice. *** *p* < 0.001 vs. CON. ^&^ *p* < 0.05, and ^&&&^ *p* < 0.001 vs. HFD. ^#^ *p* < 0.05, ^##^ *p* < 0.01, and ^###^ *p* < 0.001 vs. EA.

To assess liver fibrogenesis tendency in MAFLD, histological examination with liver Sirius Red staining was utilized and indicated amelioration of histopathologic deposition of hepatic fibrosis and collagen after EA, whereas α-BGT or deficiency of HO-1 weakened the effective effects of EA on liver fibration (Figure 3A). We also explored α-smooth muscle actin (α-SMA) content in the liver, the pathologic indicator of hepatic stellate cells contributing to hepatic fibrillogenesis. Compared with the HFD group, α-SMA content declined in the EA group. Additionally, we further examined the mRNA expression of several potent hepatic fibrogenic factors, including *collagen I*, *transforming growth factor β* (*TGF-β*), *actin α2* (*ACTA2*), and *tissue inhibitor metalloproteinase-1* (*TIMP-1*), which concluded that EA could downregulate the genetic expressions of these fibrogenic factors in MAFLD (Figure 3B–E). Overall, EA alleviated liver fibrosis advance in MAFLD and reinforced significant expression of α7nAChR and HO-1.

### 3.3. EA Restored the Homeostasis of Inflammatory Responses of MAFLD

**Figure 4:**
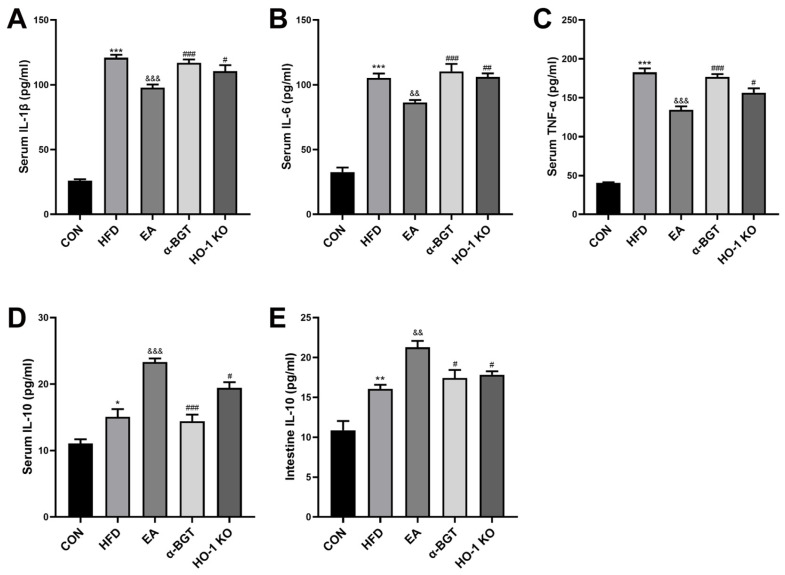
EA restored the homeostasis of systemic inflammatory responses of MAFLD. (**A**–**D**) The serum contents of IL-1β, IL-6, TNF-α, and IL-10 were evaluated by ELISA. (**E**) The intestinal content of IL-10 was evaluated by ELISA. Data were obtained from at least three independent assays in several groups of mice. * *p* < 0.05, ** *p* < 0.01, and *** *p* < 0.001 vs. CON. ^&&^ *p* < 0.01, and ^&&&^ *p* < 0.001 vs. HFD. ^#^ *p* < 0.05, ^##^ *p* < 0.01, and ^###^ *p* < 0.001 vs. EA.

Next, we investigated concentrations of inflammation-related mediators under EA intervention. As demonstrated in Figure 4A–C, compared to the CON group, increments in serum IL-6, IL-1β, and TNF-α contents were found in HFD-fed mice, suggesting the imbalance existence of systemic inflammation immunity in MAFLD. Serum contents of these pro-inflammatory indicators showed lower tendencies after EA, while α-BGT and HO-1 KO, in turn, increased concentrations of these elements. Meanwhile, as illustrated in Figure 4D,E, EA boosted the release of IL-10 in serum and intestinal tissues, an important anti-inflammatory mediator, suggesting the potential potency of EA against inflammatory responses. However, the anti-inflammation benefits of EA partly failed when treated with α-BGT or HO-1 KO. Taken together, the therapeutic advantage of EA was featured with functionally suppressive impacts on pro-inflammatory cytokine production and homeostasis maintenance in inflammation responses dependent on α7nAChR-associated HO-1 in MAFLD.

### 3.4. EA Attenuated Impaired Gut Barrier in MAFLD

As previously depicted, HFD promoted the aggravation of hepatic steatosis and fibrosis in MAFLD, but precise mechanisms remained unclear. Emerging evidence revealed that together with metabolic-related abnormalities, MAFLD was always accompanied by intestinal barrier destruction, further exacerbating MAFLD progression and systemic inflammation via the gut-liver axis, suggesting that targeting restoring gut barrier integrity could be an effective resource in MAFLD. Thus, we further explored whether EA exhibited therapeutic potential in intestinal perturbation.

**Figure 5:**
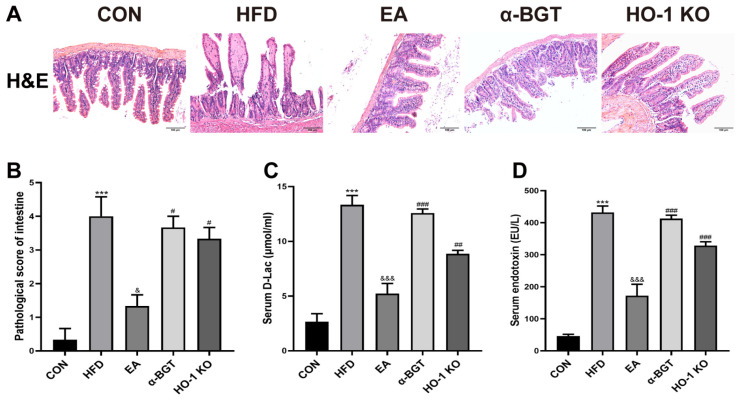
EA attenuated evident intestinal injury and decreased gut permeability in MAFLD. (**A**) H&E staining was utilized for pathological alterations in the intestine expressed in representative images (scale bar = 100 μm). (**B**) The intestinal pathological score was assessed. (**C**,**D**) Serum levels of D-Lac and endotoxin were assessed by ELISA. Data were obtained from at least three independent assays in several groups of mice. *** *p* < 0.001 vs. CON. ^&^ *p* < 0.05, and ^&&&^ *p* < 0.001 vs. HFD. ^#^ *p* < 0.05, ^##^ *p* < 0.01, and ^###^ *p* < 0.001 vs. EA.

As depicted in Figure 5A,B, morphological characteristics of intestinal H&E staining in the HFD group showed more distinct intestinal mucosal damage compared to the CON group, with manifestations of several scathed lesions in the gut villi, different degrees of partial detachment of intestinal epithelial cells, destruction or absence of crypt structures, reduction in goblet cells and infiltration of inflammatory cells, along with higher pathological scores. In addition, EA displayed protective restoration on disrupted intestinal tissues, while α-BGT or HO-1 KO received the opposite results. Furthermore, we detected the serum levels of D-Lac and endotoxin, markers of intestinal mucosa damage and permeability. As shown in Figure 5C,D, HFD-fed mice had elevated serum concentrations of D-Lac and endotoxin relative to the CON group. The EA-treated mice revealed lower D-Lac and endotoxin levels than HFD-treated mice, but the protective effects were eliminated in the α-BGT and HO-1 KO groups, implying the importance of α7nAChR-mediated HO-1 on the restoration of intestinal disruption during EA.

**Figure 6:**
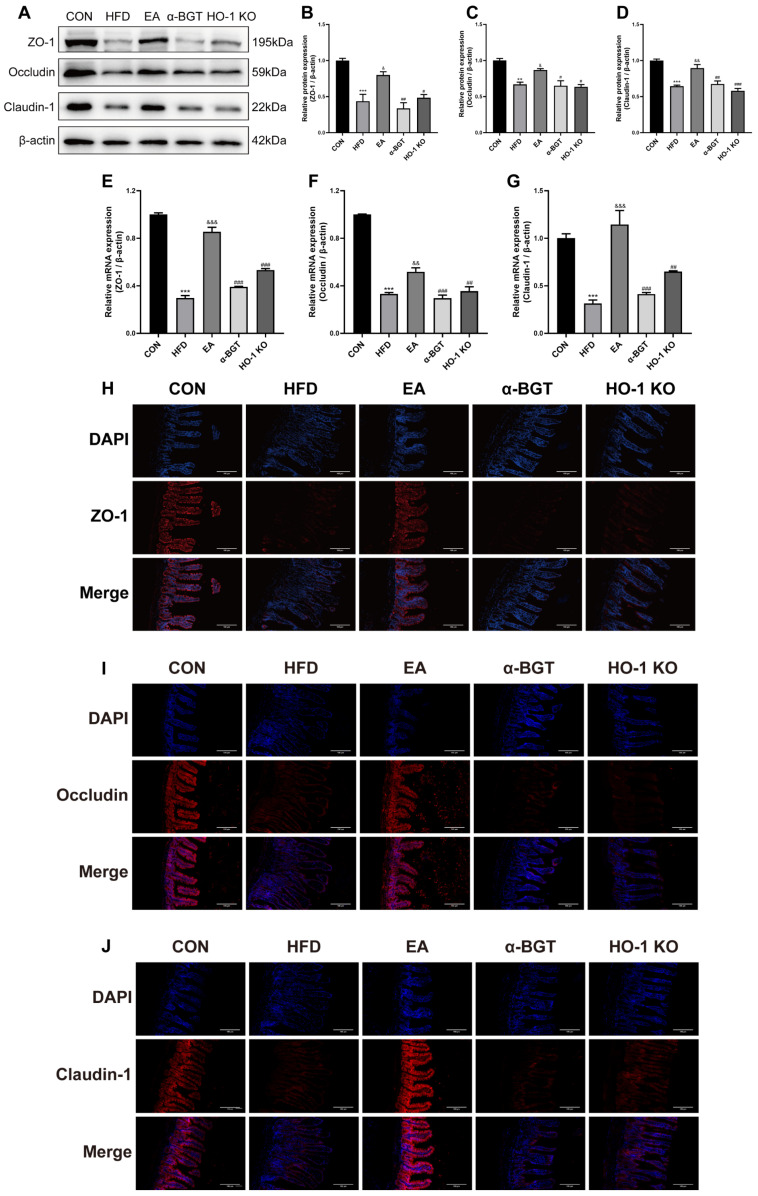
EA enhanced intestinal tight junction expression in MAFLD. (**A**–**D**) Relative protein contents of ZO-1, Occludin, and Claudin-1 were evaluated by Western blot. (**E**–**G**) Relative mRNA contents of *ZO-1*, *Occludin*, and *Claudin-1* were evaluated by RT-qPCR. (**H**–**J**) Immunofluorescence assays, respectively, with ZO-1, Occludin, and Claudin-1 (red) and DAPI (blue) (scale bar = 100 μm). Data were obtained from at least three independent assays in several groups of mice. ** *p* < 0.01, and *** *p* < 0.001 vs. CON. ^&^ *p* < 0.05, ^&&^ *p* < 0.01, and ^&&&^ *p* < 0.001 vs. HFD. ^#^ *p* < 0.05, ^##^ *p* < 0.01, and ^###^ *p* < 0.001 vs. EA.

Next, we investigated intestinal tight junction levels, which secured intestinal integrity and maintained homeostasis. Compared to the CON group, HFD elicited a significant decline in the intestinal protein and gene levels of ZO-1, Occludin, and Claudin-1, indicating accompanying disruption of the intestinal tight junctions followed by the administration of HFD (Figure 6A–G). EA upregulated the intestinal levels of tight junction proteins, whereas obvious downregulation tendencies were observed in the α-BGT and HO-1 KO groups, suggesting the compromise on the intestinal mucosal barrier. A similar tendency was also found in the immunofluorescence staining results (Figure 6H–J). Overall, EA dampened intestinal mucosal destruction and preserved the intestinal barrier homeostasis, which was in correlation with the expression of α7nAChR and HO-1.

### 3.5. The Potential Mechanism of EA Stimulation in the Mitigation of MAFLD Might Be Attributed to the Involvement of the HO-1/p38 MAPK/NF-κB Pathway Mediated by α7nAChR

The above results draw empirical attention to the key existence of α7nAChR-associated HO-1 expression during EA treatment in MAFLD. Given that p38 MAPK is a possible downstream regulatory factor of HO-1 and subsequently suppressed NF-κB-mediated inflammatory signaling to restore the immune economy, we then researched the effects of EA stimulation on the α7nAChR-related HO-1/p38 MAPK/NF-κB pathway in MAFLD.

**Figure 7:**
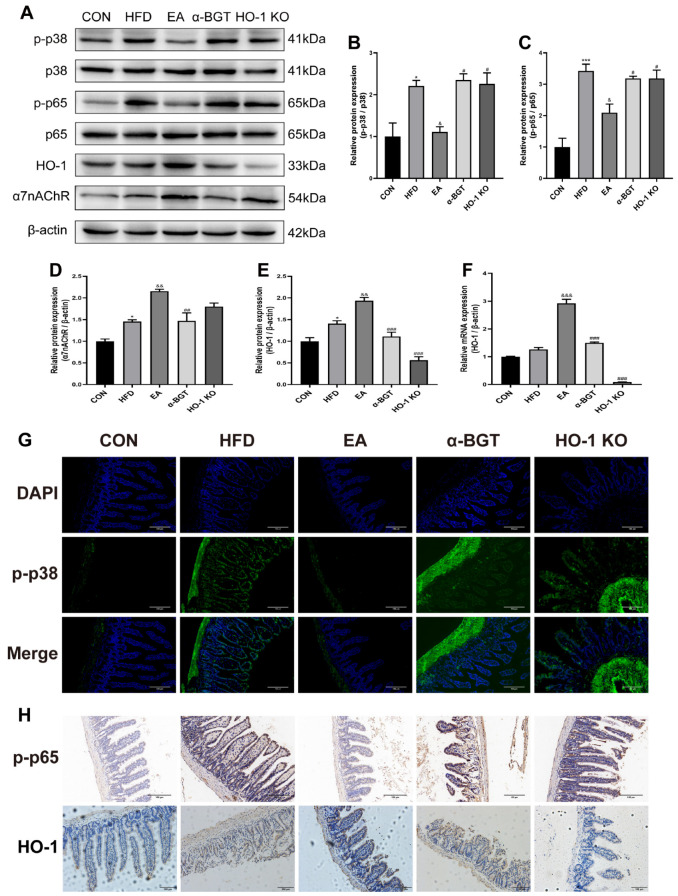
EA mitigated MAFLD via the involvement of the α7nAChR-mediated HO-1/p38 MAPK/NF-κB pathway. (**A**–**E**) Relative protein contents of p-p38 MAPK, p38 MAPK, p-p65, p65, α7nAChR, and HO-1 were evaluated with Western blot. (**F**) The relative mRNA content of *HO-1* was evaluated with RT-qPCR. (**G**) Immunofluorescence assay with p-p38 MAPK (green) and DAPI (blue) in the intestine tissues (scale bar = 100 μm). (**H**) Immunohistochemistry assays with p-p65 and HO-1 in the intestine tissues (scale bar = 100 μm). Data were obtained from at least three independent assays in several groups of mice. * *p* < 0.05, and *** *p* < 0.001 vs. CON. ^&^ *p* < 0.05, ^&&^ *p* < 0.01, and ^&&&^ *p* < 0.001 vs. HFD. ^#^ *p* < 0.05, ^##^ *p* < 0.01, and ^###^ *p* < 0.001 vs. EA.

Mice with HFD displayed augmented intestinal expressions of p-p38 MAPK and NF-κB p-p65, along with upregulated α7nAChR and HO-1 expression, compared with the CON group (Figure 7A–F). Moreover, it was evidenced that enhanced expressions of α7nAChR and HO-1, as well as declined expressions of p-p38 MAPK and p-p65, were found after EA. However, α7nAChR inhibition or HO-1 KO was involved in the enhancive activation of the MAPK/NF-κB pathway relative to the EA-treated group. Additionally, analogous effects were found in the immunofluorescence staining results for p-p38 MAPK and immunohistochemistry staining results for p-p65 and HO-1, as shown in Figure 7G,H. Overall, our results implied that the p38 MAPK/NF-κB p65 pathway exerted a vital impact on the therapeutic mechanisms of EA in MAFLD, which may be related to the enhancement of α7nAChR-mediated HO-1 expression.

## 4. Discussion

In the current investigation, we identified the therapeutic effects of EA at ST-36 via activating CAP for intestinal integrity preservation in MAFLD. The dependence on the expression of α7nAChR-associated HO-1 appeared to be a vital mechanism through which EA suppressed the p38 MAPK/NF-κB pathway to ameliorate MAFLD-related injury, indicating EA at ST-36 may be a potential therapeutic strategy for MAFLD.

Accumulating experimental evidence has demonstrated the valuable impacts of EA on the pleiotropic modulation of massive immune responses, gastrointestinal motility, and inflammatory reactions in numerous diseases [20,21,22]. Meanwhile, whether EA at ST-36 exerts protective impacts on the progression of MAFLD has not been clearly illustrated. Our previous study demonstrated that EA at ST-36 promoted the remission of liver damage in cholestatic liver injury mice [12]. In line with the previous research, we found the advantages of EA were exhibited by alleviating abnormal lipid metabolic parameters and liver damage-related indicators, reducing hepatic lipid deposition, and ameliorating liver fibrosis, suggesting that EA is therapeutic in the development of hepatic fatty degeneration and fibrogenesis in MAFLD. In addition, emerging evidence has elucidated that HFD containing enriched dietary emulsifier components may trigger low-grade systemic inflammatory cascades in mice [23,24,25], while EA demonstrates efficacy in attenuating inflammation in BDL-induced liver injury and dextran sulfate sodium (DSS)/2,4,6-trinitrobenzene sulfonic acid (TNBS)-induced colitis [12,26,27,28]. Therefore, serum concentrations of several critical inflammation-related mediators were analyzed in the current study, demonstrating that EA maintained the homeostasis of inflammation responses by downregulating the levels of several inflammatory factors that were attributed to the defective intestinal barrier and upregulating IL-10 contents. This finding provides a compelling clue that EA might be a valuable tool to attenuate MAFLD-related metabolic dysfunction via mitigation of inflammation responses.

In addition to the abovementioned dysfunctions, gut barrier leakage has been gradually emphasized as a significant constituent prerequisite in MAFLD progression [3]. The indicative characteristics of intestinal barrier dysfunction cover elevated intestinal permeability, dysregulated tight junctions, intestinal flora dysbiosis, and intestinal epithelial injury [5,29]. Recent evidence has indicated that EA poses effectiveness on impaired gut barrier in DSS-elicited colitis [26,30]. Our previous research demonstrated that EA at ST-36 preserved the gut barrier integrity in the BDL-generated liver damage model as well [12]. Therefore, EA’s effective function upon disrupted gut barrier in MAFLD was evaluated. Endotoxin is a biological type of pathogen-associated molecular pattern (PAMP), driving sustained pro-inflammatory cascades and perturbing bidirectional gut-liver communication [29,31]. Moreover, D-Lac is a bacterial metabolite that enters the bloodstream under pathological intestinal permeability damage [32]. In this recent study, dramatic decreases in the serum D-Lac and endotoxin levels were observed in the EA group, emphasizing the effectiveness of EA in improving disordered intestinal barrier permeability. Additionally, there was a notably enriched expression of tight junction proteins after EA, which implied enhanced enrichment of essential regulators in epithelial defense mechanisms. Overall, the results confirmed the curative roles of EA at ST-36 in protecting intestinal epithelial cells from pathologic conditions and preserving intestinal barrier integrity.

Increasing evidence suggests that EA is important in the intricate interplay between neural and immune systems, exerting potent effects dependent on activating vagal nerves, thereby inducing acetylcholine (ACh) neurotransmitter release that subsequently interacts with α7nAChR [33,34]. α7nAChR, a critical regulatory receptor mostly expressed on the surface of various immune cells, instantly reacts to EA stimulation and mediates inflammation suppression and immune homeostasis maintenance, which was recognized as CAP [33,35]. Emerging research indicates that α7nAChR can mediate anti-inflammatory and immunomodulatory potencies of vagal nerve stimulation by attenuating intestinal permeability, suppressing abnormal bacterial translocation, strengthening gut tight junction molecules, and restoring the inflammation-related balance in the colitis models [36,37]. In the recent investigation, EA at ST-36 significantly enhanced the expression of α7nAChR. However, the administration of α-BGT targeting the inhibition of α7nAChR exactly abolished EA’s protective effects, reinforcing that α7nAChR might participate in the immunomodulation of EA in the intestine.

HO-1, with biological anti-oxidant and anti-inflammatory properties, is upregulated and exhibits immunomodulatory functions in response to massive stress stimulation. The cytoprotective role of HO-1 has been emphasized through studies relying on the special cells and animals with HO-1 knockdown, which also poses increasing relevance in oxidative stress and diseases such as tetrachloromethane (also called CCl_4_)-induced liver injury [38,39]. Recent evidence indicates that the activation of α7nAChR by EA stimulation or specific pharmacological agents can upregulate HO-1 expression, thereby reducing intestinal mucosal damage, restoring intestinal permeability, and enhancing tight junction protein expressions in BDL-induced liver injury [12,16]. Consistent with prior findings, we found that EA at ST-36 activated α7nAChR and induced HO-1 upregulation. Notably, deficient intestinal HO-1 rendered EA-mediated protective effects against liver steatosis and fibrosis, intestinal barrier disruption, and inflammation. Furthermore, α7nAChR inhibition reduced HO-1 expression and eliminated EA efficaciousness, highlighting the essential significance of HO-1 being involved in the EA through α7nAChR-related signaling pathways in MAFLD.

**Figure 8:**
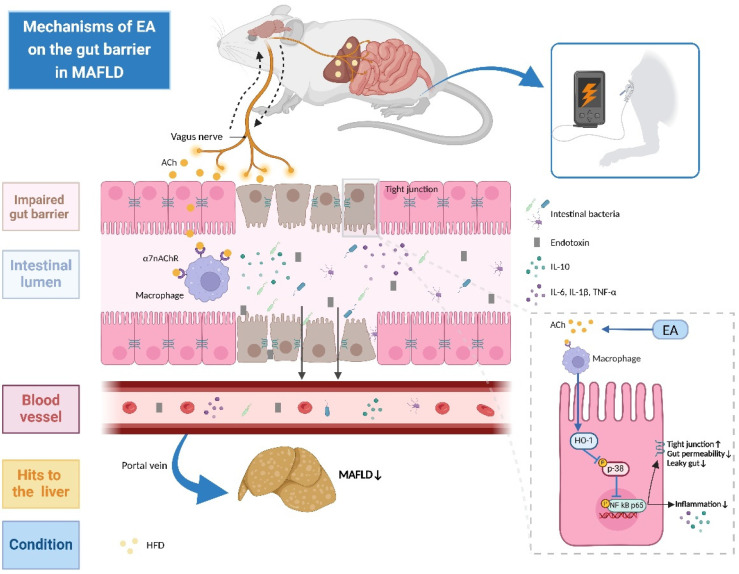
Schematic presentation of the underlying mechanisms of EA at ST-36 on the gut barrier in MAFLD. EA treatment activates the vagus nerve-mediated CAP, in which ACh released by vagal efferent fibers binds to α7nAChR on macrophages to promote anti-inflammation effects. It enhances HO-1 expression, thereby suppressing the phosphorylation of downstream targets p38 MAPK and NF-κB p65, ultimately repairing gut leakage (including enhanced expressions of tight junctions and decreased gut permeability) and reducing inflammation to ameliorate the pathogenesis of HFD-induced MAFLD via the gut–liver axis.

Furthermore, we discovered the underlying mechanisms of how EA at ST-36 exerted protective effects in MAFLD. The MAPK pathway is a pleiotropic transducer mediating numerous extracellular signals to preserve immunity homeostasis in several pathophysiological processes such as inflammation, immune responses, apoptosis, oxidative stress, and tumorigenesis; additionally, p38 MAPK is one of the most significant subfamilies [40]. The p38 MAPK signaling has been reported to exert a vital role in restoring intestinal barrier integrity [41,42]. Moreover, our previous investigation suggested that the induction of HO-1 preserved gut barrier permeability by inhibiting the activation of p38 MAPK, whereas the suppression of HO-1 reversely promoted the phosphorylation of p38 MAPK with the worsened intestinal barrier in both Caco-2 cells and CCl_4_-induced liver damage mice, indicating the crucial role of HO-1 in modulating the p38 MAPK signaling [38]. In addition, once activated, p38 MAPK functions as a vital transducer molecule in a variety of cellular responses, thereby triggering the following downstream cascades of the NF-κB signaling pathway [43]. NF-κB p65 served as a key transcription molecule by evoking the secretion burst of various inflammatory cytokines, further exacerbating the progression of numerous diseases, such as colitis and BDL-induced liver injury [12,41,44]. Consistent with previous investigations, we demonstrated that EA at ST-36 eased intestinal phosphorylated enrichment of p38 MAPK and NF-κB p65 in MAFLD. However, α-BGT administration that suppressed α7nAChR expression or HO-1 deficiency further promoted the biological activation of p38 MAPK and NF-κB p65, confirming the involvement of the α7nAChR-dependent HO-1/p38 MAPK/NF-kB axis in combating the pathogenesis of the MAFLD-accompanied intestinal barrier impairment via EA stimulation at ST-36, as illustrated in Figure 8. These results reveal a novel perspective for potential modulatory mechanisms in the progression of MAFLD and provide an alternative therapeutic option for the treatment of MAFLD.

## 5. Limitations

Several limitations should be drawn in the present research. First, given that estrogen has been shown to modulate lipid metabolism in MAFLD [45,46], this study was conducted exclusively in male mice to control for cyclic hormonal fluctuations. Future investigations comparing both sexes or hormone-manipulated models are warranted to elucidate potential sex-dependent therapeutic outcomes of EA. Second, possible alterations in gut flora dysbiosis might also impact the progression of MAFLD; therefore, further investigations into intestinal microbiome imbalance could be conducted. Third, multi-omics integrated analysis could be considered for additional verification evidence of the therapeutic potency of EA in MAFLD at the molecular and cellular levels, along with further exploration of other potential mechanisms. Fourth, our investigation suggested that EA might be a safe and effective therapeutic strategy for MAFLD with clinical translational potential. Nevertheless, further clinical investigations are warranted to validate the efficacy of EA in patients.

## 6. Conclusions

In this study, we confirmed that EA at ST-36 ameliorated MAFLD-induced abnormalities in the intestinal barrier, confirming the effective potential of EA. The underlying molecular mechanisms of protective effects of EA revealed the significant participation of the p38 MAPK/NF-kB signaling pathway, in which α7nAChR-mediated HO-1 simultaneously plays a critical role. Collectively, our study highlighted the promising therapeutic strategy of EA at ST-36, paving the way for the optimization of non-pharmacological and painless interventions for MAFLD.

## Figures and Tables

**Table 1:** The specific primers.

Genes	Specific Primer Sequences
*Collagen I*	Forward: ACAGGCGAACAAGGTGACAGAG Reverse: AGGAGAACCAGGAGAACCAGGAG
*TGF-β*	Forward: CAACAATTCCTGGCGTTACCTTGG Reverse: TGTATTCCGTCTCCTTGGTTCAGC
*ACTA2*	Forward: ATCAGGGAGTAATGGTGGAATGGG Reverse: CAGTTGGTGATGATGCCGTGTTC
*TIMP-1*	Forward: GGATTCAAGGCTGTGGGAAATGC Reverse: TTCACTGCGGTTCTGGGACTTG
*ZO-1*	Forward: TATGGCTTGTGGGGTGTT Reverse: GGCTAGGTGTTTGGGGAT
*Occludin*	Forward: CTGCCTGCACGATGTCT Reverse: GAGTGTTCAGCCCAGTCAA
*Claudin-1*	Forward: GCCTGCAAGAGGGATGT Reverse: GGGATGATAGTGCCCAGTC
*HO-1*	Forward: ACAGCCCCACCAAGTTC Reverse: GGCGGTCTTAGCCTCTTC
*β-actin*	Forward: GATGGTGGGAATGGGTCAGAAGG Reverse: TTGTAGAAGGTGTGGTGCCAGATC

**Table 2:** The alterations of body weight and visceral fat index in several groups.

Measurement	Age (Week)	CON	HFD	EA	α-BGT	HO-1 KO
Baseline body weight (g)	8	19.72 ± 0.24	19.85 ± 0.14	19.94 ± 0.15	20.00 ± 0.21	19.96 ± 0.20
Ultimate body weight (g)	20	28.50 ± 0.43	35.73 ± 0.46 ***	29.84 ± 0.36 ^&&&^	35.01 ± 0.58 ^###^	32.58 ± 0.25 ^##^
Visceral fat index (%)	20	2.46 ± 0.19	5.55 ± 0.15 ***	3.70 ± 0.17 ^&&&^	5.10 ± 0.20 ^###^	4.93 ± 0.18 ^###^

Data are shown as mean ± standard error and achieved from at least three independent assays in several groups of mice. *** *p* < 0.001 vs. CON. ^&&&^ *p* < 0.001 vs. HFD. ^##^ *p* < 0.01, and ^###^ *p* < 0.001 vs. EA.

## Data Availability

The data supporting the conclusions of this article are available from the corresponding author upon reasonable request. The data are not publicly available due to privacy.
